# The relationship between bile acid concentration, glucagon-like-peptide 1, fibroblast growth factor 15 and bile acid receptors in rats during progression of glucose intolerance

**DOI:** 10.1186/s12902-017-0211-5

**Published:** 2017-09-25

**Authors:** Xinfeng Yan, Peicheng Li, Zhaosheng Tang, Bo Feng

**Affiliations:** Department of Endocrinology, Shanghai East Hospital, Tongji University School of Medicine, 150 Jimo Road, Shanghai, 200120 China

**Keywords:** Impaired glucose tolerance, Type 2 diabetes mellitus, Bile acid receptors, GLP-1, FGF15

## Abstract

**Background:**

Recent studies show that bile acids are involved in glucose and energy homeostasis through activation of G protein coupled membrane receptor (TGR5) and farnesoid X receptor (FXR). A few researches have explored changes of TGR5 and FXR in animals with impaired glucose regulation. This study aimed to observe changes of plasma total bile acids (TBA), glucagon-like-peptide 1 (GLP-1), fibroblast growth factor 15 (FGF15), intestinal expressions of TGR5 and FXR, and correlations between them in rats with glucose intolerance.

**Methods:**

Besides plasma fasting glucose, lipid, TBAs, alanine transaminase (ALT), active GLP-1(GLP-1A) and FGF15, a postprandial meal test was used to compare responses in glucose, insulin and GLP-1A among groups. The expressions of TGR5 and FXR in distal ileum and ascending colon were quantified by real-time PCR and western blot.

**Results:**

TGR5 expression was significantly decreased in distal ileum in DM group compared to other groups, and TGR5 and FXR expressions in ascending colon were also decreased in DM group compared to other groups. Correlation analysis showed correlations between TBA and GLP-1A or FGF15. GLP-1A was correlated with TGR5 mRNA expression in colon, and FGF15 was correlated with FXR mRNA expression in colon.

**Conclusions:**

These results indicates that bile acid-TGR5/FXR axis contributes to glucose homeostasis**.**

## Background

Bile acids have been known mainly for their key physiological role in dietary fat absorption and cholesterol excretion [1]. In 1999, bile acids were discovered to be natural ligands of the nuclear receptor, FXR [2],which is highly expressed in liver, intestine, kidney, and adrenal glands. The FXR pathway is important in negative feedback regulation of bile acid synthesis. FXR in liver is activated by bile acids synthesized in the hepatocytes or from the enterohepatic circulation to inhibit expression of cholesterol 7ɑ-hydroxylase (CYP7A1), a rate-limiting enzyme in bile acid synthesis. Another FXR signaling pathway that suppresses hepatic bile acid synthesis involves fibroblast growth factor 19 (FGF19, rodent ortholog is FGF15) [3, 4], which is produced following activation of FXR by bile acids in the distal ileum. By binding to FGF receptor 4 (FGFR4) in liver, FGF19 represses CYP7A1 expression to inhibit bile acid synthesis.

The second major bile acid receptor raised interest in recent years is the G protein coupled membrane receptor (GPCR), TGR5, also known as G protein-coupled bile acid receptor 1 (GPBAR 1) [5]. It has been demonstrated that bile acids contribute to GLP-1 secretion through TGR5 in STC-1, a murine enteroendocrine cell line [6]. GLP-1 can stimulate insulin secretion and suppress glucagon secretion in a glucose-dependent manner and regulate glucose homeostasis [7, 8]. The importance of bile acid-TGR5-GLP-1 pathway was shown in a series of studies in vivo and cell culture by Thomas et al. in 2009 [9] using mice with targeted disruption and overexpression of the TGR5 gene.

Based on the regulation of bile acid on lipid and glucose homeostasis, we hypothesized that an alteration of bile acid-FXR/TGR5 pathway may contribute to the progression of glucose intolerance. Little is known about changes of TGR5 and FXR in animals with impaired glucose regulation. In this study, we assessed whether there were correlations between plasma TBA, GLP-1, FGF15, and expressions of TGR5 and FXR in intestine during progression of glucose intolerance in rats.

## Methods

### Drugs and diets

Regular chow (containing 15% fat and 65% carbohydrate by energy, provided energy 16.1KJ/g) and high-fat, high-sugar diet (containing 40% fat and 40% carbohydrate by energy, provided energy 18.7KJ/g) was purchased from Slaccas Laboratory Animal,Shanghai, China. The Streptozotocin (STZ) was purchased from Sigma-Aldirch Co., St.Louis, USA. The dipeptidyl peptidase-4 (DPP4) inhibitor was purchased from Millipore, St. Charles, USA.

### Animal experiments

Thirty-three four-week old male SD rats (pathogen-free grade, average body weight 110 g) were purchased from Slaccas Laboratory Animal, Shanghai, China. They were divided into 3 groups: 1) Normal Control (NC) group, 10 rats were fed with regular chow; 2) Impaired glucose tolerance (IGT) group, 10 rats were fed with high-fat, high-sugar diet serving as insulin-resistant models; 3) Type 2 diabetes mellitus (DM) group, 13 rats were treated additionally with a low dose of STZ after four-week high-fat, high-sugar diet to induce type 2 diabetes. All rats were fed 7 days for environment adaptation. Then the rats in the IGT and DM groups were fed with high-fat, high-sugar diet for 4 weeks. After fasting for 12 h, an OGTT test was performed in the groups to confirm insulin resistance. The rats were fed with 50% D-glucose solution at 2 g/kg body weight via oral gavage, and tail venous blood glucose was tested at 0 min, 30 min, 60 min, 90 min and 120 min. The day after OGTT test, the rats in DM group were intraperitoneally injected with STZ (30 mg/kg body weight) to induce type 2 diabetes [10], and the rats in NC and IGT group were intraperitoneally injected with the same dose of sodium citrate. Blood glucose was tested 72 h after STZ was injected, and rats with fasting plasma glucose (FPG) >11.1 mmol/L were considered to have developed type 2 diabetes [11, 12]. 1 rats died and 2 rats failed to develop diabetes. Then these rats were raised for 12 weeks, body weight and glucose were monitored every week.

To observe the postprandial response of plasma glucose, insulin and GLP-1A in these rats, a postprandial test [13] was performed after 12-h fasting (from 8 PM to 8 AM). At 8 AM all the rats were fed diet like usual for 2 h and then the diets were removed. Blood samples were collected from the tail immediately prior (at 0) and at 1, 2, 3 and 4 h after the initiation of diet feeding. Thus blood samples at 3 and 4 h reflected the changes of plasma glucose, insulin and GLP-1A 1or 2 h after the meal. Total bile acids at the 5-h time point were measured only in the NC group.

All rats were sacrificed at the end of experiment. Under anesthesia with intraperitoneally administered pentobarbital sodium, blood was collected with Na-heparin injectors into Na-heparin tubes from the heart left ventricles. Subsequently, the blood was centrifuged and the plasma was separated for determination of glucose, TBA, ALT, total cholesterol, triglyceride, GLP-1A and FGF15 levels. The distal region of ileum and ascending colon were collected. The mucosa of distal ileum and ascending colon were collected and frozen immediately in liquid nitrogen for measurement of mRNA and protein expression of TGR5 and FXR in the intestine.

### Measurement of glucose, TBA, ALT, insulin, FGF15 and GLP-1A

Plasma levels of glucose, ALT and TBA were determined by automatic biochemical analyzer (Roche Cobas 8000). Plasma insulin were measured by Rat Insulin Elisa kit (Millipore, St. Charles,USA). FGF15 was determined using FGF15 Elisa Kit (USCN Life Science Inc., Wuhan, China) for rats. GLP-1A was determined using GLP-1 RIA kit (Millipore, St. Charles,USA) according to the instructions of the manufacturer. For testing GLP-1A, the blood samples were collected in ice-cooled EDTA-tubes with a DPP4 inhibitor, and then centrifuged at 4 °C. Sample plasma specimens were extracted with 95% ethyl alcohol. After the alcohol was evaporated, the samples were rehydrated. GLP-1A antibody was then added and incubated overnight at 4 °C. ^125^I–GLP-1 was added to label the bound GLP-1A, which was then precipitated with carrier Ig and precipitating reagent. After centrifuging the samples, the ^125^I–GLP-1A in the pellet was counted using the Auto-Gamma (Cobra).

### Measurement of TGR5 and FXR mRNA expressions

Rats TGR5 and FXR mRNA expressions were quantitatively determined by real-time PCR. Total RNA was isolated using TRIzol reagent (Invitrogen) according to the manufacturer’s instructions. For quantitative RT-PCR analysis, 1 μg total RNA was transcribed to cDNA using the high cDNA capacity reverse transcription kit (Fermentans). All reactions were performed in triplicate in a ABI 7500 Real Time PCR System using the DNA-binding dye SYBER Green I for the detection of PCR products, with thermal cycling conditions as follows: 2 min at 50 °C and 10 min at 95 °C, followed by 40 cycles at 95 °C for 15 s and 60 °C for 1 min. Relative mRNA levels were calculated by the comparative threshold cycle method using GAPDH as the internal standard. The data was analyzed using 2^-∆∆Ct^ [14]. The primers of each target gene were: GAPDH: 5’AGTGCCAGCCTCGTCTCATAG3’, 5’CGTTGAACTTGCCGTGGGTAG3’;TGR5:5′ CTCATCGTCATCGCCAACCT3’, 5’CCATGCACCAGCAGCAGATT 3′; FXR: 5′ GGGCAACTGCGTGATGGATA3’, 5’CCCTGCATAGCTTGGTCGTG 3′.

### Western blot analysis

Western blot analysis was carried out as previously described [15]. Perfused intestine tissue was immediately frozen in liquid nitrogen, and 30 mg of intestine tissue was homogenized with Radio Immunoprecipitation Assay (RIPA) Lysis buffer. The supernatant was collected for protein quantification after centrifugation at 13,000 rpm. Proteins were transferred onto a Nylon membrane after electrophoresis and blocked with 5% skimmed milk in Tris-buffered saline solution containing 0.1% Tween 20. Membranes were incubated with rabbit anti-GPCR TGR5 (Abcam, 1:1000), rabbit anti-FXR1(Abcam, 1:200) and mice anti-GAPDH(Boster, 1:2000). HRP marked goat anti-rabbit and goat anti-mice horseradish peroxide-conjugated antibody (Jackson, 1:2000) was used as a secondary antibody, and blots were developed with chemiluminescence detection.

### Statistical analysis

2^**-∆∆CT**^ was used in the RT-PCR analysis. Data was analyzed using the SPSS13.0 statistical package. Results were expressed by the mean ± SD. One-way ANOVA followed by the least significant difference calculation or Student’s t-test was used to analyze differences between groups. Pearson and Spearman correlation coefficient (r) was used to assess associations between variables. A two-tailed test was used in all analyses. The value of significance was set at α = 0.05.

## Results

**Fig. 1:**
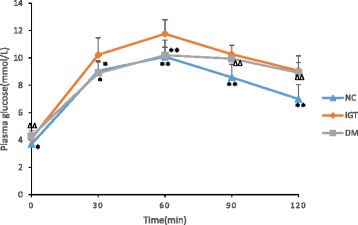
OGTT test. Compared with IGT group:* *P* < 0.05, **P < 0.01. Compared with NC group: ∆*P* < 0.05, ∆∆*P* < 0.01(*n* = 10/group)

At the 4th week, the FPG in IGT group was significantly higher than that in NC group (4.3 ± 0.4 mmol/L vs. 3.7 ± 0.3 mmol/L, *P* < 0.01). Blood glucose level at 120 min during OGTT was 8.9 ± 1.2 mmol/L which should be considered IGT by definition [16] (Fig. 1).The rats in DM group were also induced IGT successfully after four-week high-fat, high-sugar diet.

### High-fat, high-sugar diet lead to liver injury

As shown in Table 1(the plasma was collected on the day rats were sacrificed), high-fat, high-sugar diet resulted in significant increase of body weight in the IGT and DM groups compared to the NC group. FPG, cholesterol, triglyceride and ALT in IGT and DM groups were also significantly higher than the NC group. TBA level in the DM group was markedly increased compared to the NC and IGT group. There was no markedly change in FGF15 protein level in the NC group as compared with IGT group,while significantly higher than that in the DM group.

**Table 1:** Body weight and biochemical indexes

	NC	IGT	DM
Body weight (g)	465.2 ± 22.92	578.3 ± 49.89^∆∆^	560.9 ± 32.78^∆∆^
Blood glucose (mmol/L)	4.04 ± 0.26	4.8 ± 0.23^∆∆^	12.42 ± 0.7^∆∆^
Cholesterol (mmol/L)	1.28 ± 0.21	1.97 ± 0.95^∆^	2.40 ± 0.26^∆∆^
Triglyceride (mmol/L)	0.71 ± 0.22	3.25 ± 2.18^∆∆^	2.07 ± 0.59^∆^
Total Bile Acid (umol/L)	135.33 ± 23.33	135.04 ± 8.36	271.47 ± 27.49^∆∆^
FGF15 (pg/ml)	262.4 ± 21.2	275.6 ± 21.1	231.7 ± 15.8^∆∆^
ALT (mmol/L)	41.4 ± 3.8	51.5 ± 2.4^∆∆^	81.7 ± 5.6^∆∆^

Compared with NC group: ∆ *P* < 0.05, ∆∆ *P* < 0.01. Data are expressed as mean **±** SD (*n* = 10/group)

### Changes of glucose, insulin and GLP-1 in meal test

**Fig. 2:**
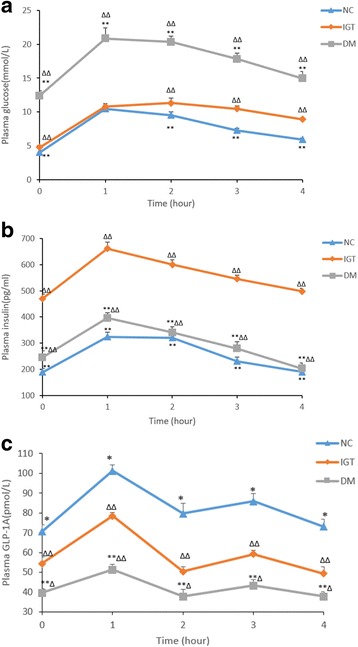
Postprandial changes in glucose (**a**), insulin (**b**) and GLP-1A (**c**). Data are represented as mean ± SD (n = 10/group). Compared with IGT group: **P < 0.01. Compared with NC group: ∆∆*P* < 0.01

Figure 2 summarized changes in the postprandial plasma glucose, insulin, and GLP-1A levels in the three groups of rats. During the meal test, the diet was provided to the rats only during the first 2 h. Figure 2a showed the changes of plasma glucose, the area under the curve (AUC) of glucose in the NC group (32.3 ± 1.4 mmol/L∙h) was 20% less (*P* < 0.01) than the IGT group (39.5 ± 1.1 mmol/L∙h), and 66% less (*P* < 0.01) than the DM group(72.7 ± 3.0 mmol/L∙h). Figure 2b demonstrated that the plasma insulin level in the IGT and DM group was significantly higher than the NC group (*P* < 0.01). Figure 2a and b indicated successfully modeling of insulin resistance and type 2 diabetes. Figure 2c showed GLP-1A changed markedly during the meal test in the three groups, the GLP-1A levels in the DM group were significantly lower than the NC and IGT groups (*P* < 0.01).

### mRNA expressions of TGR5 and FXR decreased in DM group

**Fig. 3:**
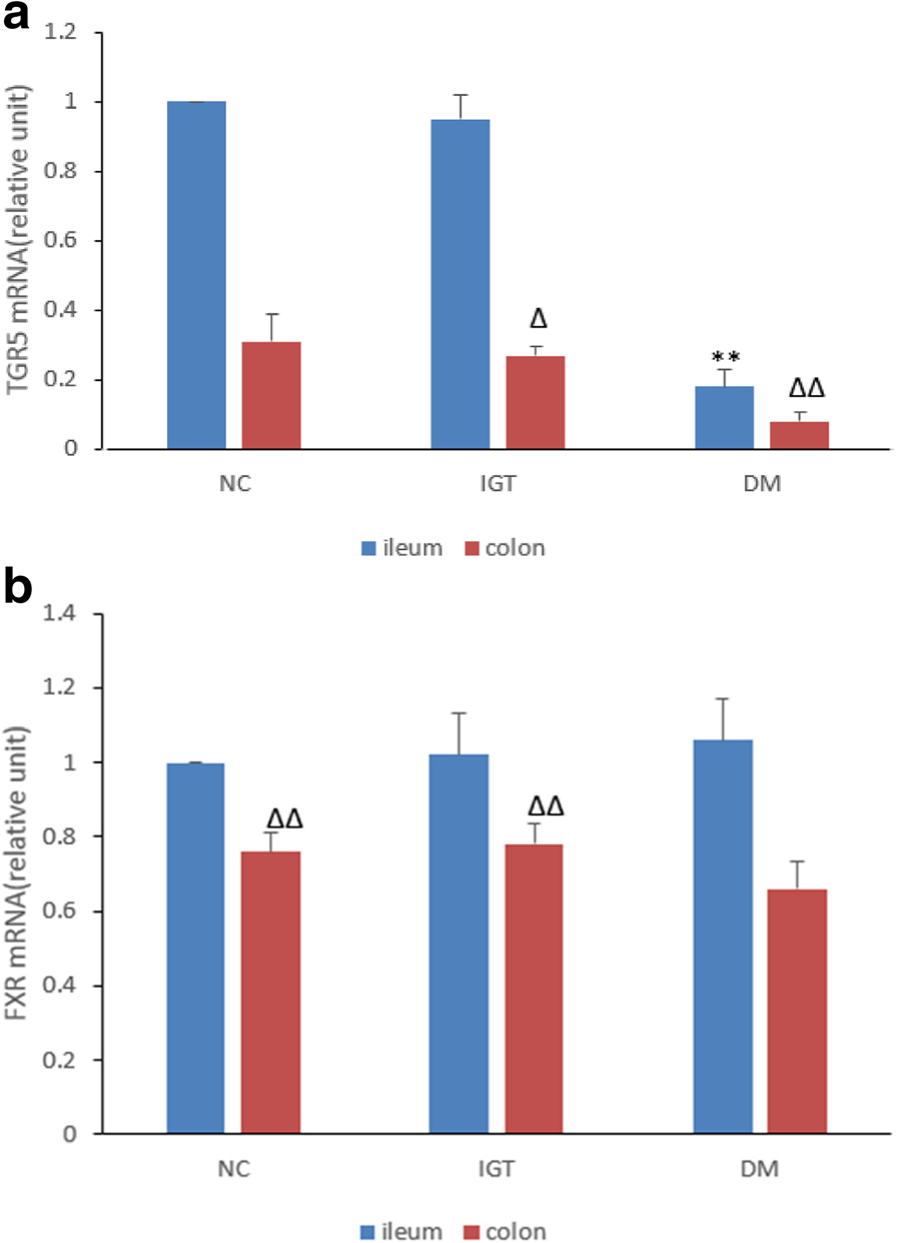
**a** TGR5 mRNA expression in distal ileum and ascending colon. Compared with NC and IGT ileum group: **P < 0.01; Compared with NC colon group: ∆*P* < 0.05, ∆∆*P* < 0.01.The data (relative unit) are shown as means ± SD (*n* = 10/group), the percentage change compared with the value of TGR5 mRNA in the NC group in the ileum. **b** FXR mRNA expression in distal ileum and ascending colon. Compared with DM colon group: ∆∆*P* < 0.01. The data (relative unit) are shown as means ± SD (*n* = 10/group), the percentage change compared with the value of FXR mRNA in the NC group in the ileum

The mRNA expressions of TGR5 and FXR in the mucosa of distal ileum and ascending colon in the three groups was summarized in Fig. 3. Figure 3a demonstrated that TGR5 mRNA expression in ileum and colon in DM group was significantly decreased compared with NC and IGT groups. There was no significant difference of TGR5 mRNA expression in ileum between NC and IGT group. Figure 3b demonstrated that FXR mRNA expression in colon in DM group was significantly decreased compared with both NC and IGT group (*P* < 0.01). No difference of FXR mRNA expression in ileum was observed among the three groups.

### Correlation analysis

**Fig. 4:**
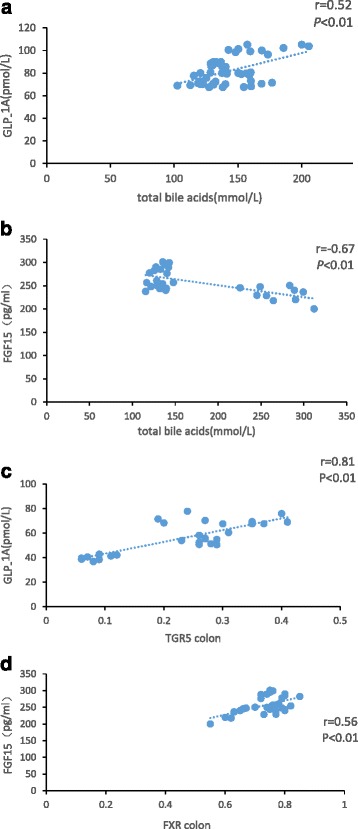
Scatter graph demonstrating the correlation between (**a**) total bile acids and GLP-1; (**b**) total bile acids and FGF15; (**c**)expression of mRNA of TGR5 in colon and GLP-1; (**d**) expression of mRNA of FXR in colon and FGF15

Correlation analysis between plasma TBA, GLP-1A, FGF15, TGR5 and FXR mRNA expressions was performed(Fig. 4). TBA was significantly positively correlated with GLP-1A (*r* = 0.52, *P* < 0.01), while negatively correlated with FGF15 (*r* = −0.67, *P* < 0.01). GLP-1A was also significantly correlated with TGR5 mRNA expression in colon (*r* = 0.81, *P* < 0.01). FGF15 was significantly correlated with FXR mRNA expression in colon (*r* = 0.56, *P* < 0.05), but without correlation in ileum. No correlation was found between GLP-1A levels and FXR mRNA expression or FGF15 levels and TGR5 mRNA expression.

### Western blot

**Fig. 5:**
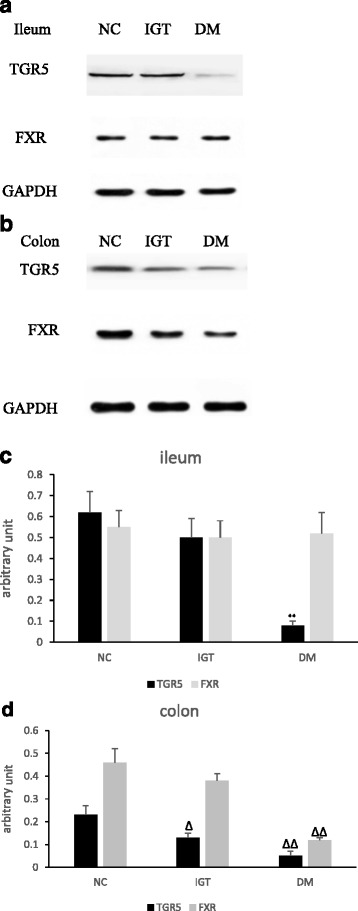
Expression of TGR5 and FXR in ileum and ascending colon. **a** Western blot analysis for TGR5 and FXR in ileum in three groups. **b** Western blot analysis for TGR5 and FXR in ascending colon in three groups. **c** densitometry readings comparing the samples shown in A, normalized to GAPDH. ** *P* < 0.01, DM vs. NC or IGT group. D:densitometry readings comparing the samples shown in B, normalized to GAPDH. The data (arbitrary unit) are shown as means ± SD (*n* = 10/group). ∆ *P* < 0.05, IGT vs. NC, ∆∆ *P* < 0.01 DM vs. NC

We quantified protein levels of TGR5 and FXR in both ileum and colon (Fig. 5). We found that there was decreased protein expression of TGR5 in the DM group compared with NC group in both ileum and colon. No differences in protein expression of FXR in distal ileum were found among groups, while the protein expression of FXR in ascending colon in DM group was markedly decreased than NC and IGT groups.

## Discussion

The present study demonstrated changes of GLP-1, FGF15, bile acid receptors TGR5 and FXR in rats with different blood glucose levels, and the correlations between them. Figure 1 showed the successful modeling of IGT in the IGT and DM groups after 4 weeks of high-fat, high-sugar diet. And this is the base of successful modeling of T2DM before intraperitoneally injection of STZ. We found the TBA level increased in the DM group which was considered to be related with increased cholesterol levels and lipid-induced liver injury demonstrated by elevated ALT, while no significant change in TBA level was observed in the IGT group with elevated ALT, so we assumed that some factors besides cholesterol and liver injury affected the synthesis of bile acid. Decreased FGF15 in the DM group and the negative correlation found between TBA levels and FGF15 levels (*r* = −0.51, *P* < 0.01) suggested that decreased FGF15 leaded to increased synthesis of bile acids. This is consistent with the theory that synthesis of bile acid is inhibited by suppression of CYP7A1, which is downregulated by combination of FGF15 to the FGFR in hepatocytes [17]. Studies in mice showed that activation of intestinal FXR by bile acids induces the secretion of FGF15 [3]. In the study, we found the expression of FXR was decreased in ascending colon in DM group, and was positively correlated with FGF15 (*r* = 0.42, *P* < 0.05), so we assumed that due to decreased expression of intestinal FXR, the FGF15 was decreased leading to attenuated negative feedback regulation of bile acid synthesis. No difference was found in the ileum.

In our study, positive correlation between GLP-1A and TBA was found in NC group (*r* = 0.61, *P* < 0.01), and GLP-1A was also positively correlated with TGR5 expression in both distal ileum (*r* = 0.92, *P* < 0.01) and ascending colon (*r* = 0.72, *P* < 0.01), suggesting that the bile acids playing important roles in stimulating TGR5 in L-cells to secrete GLP-1.

Postprandial meal test showed significantly decreased plasma insulin level in the DM group, and lower GLP-1A level in both IGT and DM groups. The expression of TGR5 mRNA decreased in both IGT and DM group in distal ileum and ascending colon, and TGR5 mRNA is lower in ascending colon than distal ileum in the same group. Studies have proved that GLP-1 secretion is stimulated by activation of TGR5 expressed in enteroendocrine cells by multiple bile acids [9, 18]. Besides, Trabelsi MS, et al. [19] found that FXR inhibits GLP-1 secretion by decreasing glycolysis, which is inconsistent with our finding. We assumed that due to the decreased TGR5 mRNA expression in intestine, the GLP-1 level was still decreased though the total bile acids were increased and FXR was decreased (significantly in the colon) in the DM group.

Bile acids are known to be involved in enteroendocrine cells function and associated with energy expenditure in brown adipose tissue and muscle via stimulation of type 2 iodothyronine deiodinase [6, 20, 21]. Recent studies discovered a relationship between bile acids, bile acid receptors and gut microbiota [22, 23], which helped us to understand the role of bile acids and gut microbiota in regulating glucose and lipid. Our study just observed changes of bile acid-TGR5/FXR axis in rats during progression of glucose intolerance, further work used agonist or inhibitor of bile acids receptors would be required to identify these changes.

## Conclusion

Expression of TGR5 and FXR in intestine decreased in rats during progression of glucose intolerance. There were correlations between TBA, GLP-1, FGF15 and bile acids receptors, which might be a new target of treatment for type 2 DM in the future.

## Abbreviations

ALT: alanine transaminase
AUC: area under the curve
CYP7A1: cholesterol 7ɑ-hydroxylase
DM: diabetes mellitus
DPP-4: dipeptidyl peptidase-4
FGF15: fibroblast growth factor 15
FGFR4: fibroblast growth factor receptor 4
FPG: fasting plasma glucose
FXR: farnesoid X receptor
GLP-1: glucagon-like-peptide 1
GLP-1A: active glucagon-like-peptide 1
GPBAR1: G protein-coupled bile acid receptor 1
IGT: imparied glucose tolerance
NC: normal control
STZ: streptozotocin
TBA: total bile acids
TGR5 or GPCR: G protein coupled membrane receptor
